# Triacontanol modulates salt stress tolerance in cucumber by altering the physiological and biochemical status of plant cells

**DOI:** 10.1038/s41598-021-04174-y

**Published:** 2021-12-30

**Authors:** Mubeen Sarwar, Sumreen Anjum, Qurban Ali, Muhammad Waqar Alam, Muhammad Saleem Haider, Wajid Mehboob

**Affiliations:** 1 Department of Horticulture, University of the Punjab, Lahore, Pakistan; 2 Department of Horticulture, University of Agriculture, Faisalabad, Sub-Campus Depalpur, Okara, Pakistan; 3 Institute of Botany, University of the Punjab, Lahore, Pakistan; 4 Institute of Molecular Biology and Biotechnology, The University of Lahore, Lahore, Pakistan; 5 Department of Plant Pathology, University of Okara, Okara, Pakistan; 6 Department of Plant Pathology, University of the Punjab, Lahore, Pakistan; 7 Plant Physiology Division, Nuclear Institute of Agriculture Tando Jam, Tando Jam, Pakistan

**Keywords:** Ecology, Agroecology

## Abstract

Cucumber is an important vegetable but highly sensitive to salt stress. The present study was designed to investigate the comparative performance of cucumber genotypes under salt stress (50 mmol L^−1^) and stress alleviation through an optimized level of triacontanol @ 0.8 mg L^−1^. Four cucumber genotypes were subjected to foliar application of triacontanol under stress. Different physiological, biochemical, water relations and ionic traits were observed to determine the role of triacontanol in salt stress alleviation. Triacontanol ameliorated the lethal impact of salt stress in all genotypes, but Green long and Marketmore were more responsive than Summer green and 20252 in almost all the attributes that define the genetic potential of genotypes. Triacontanol performs as a good scavenger of ROS by accelerating the activity of antioxidant enzymes (SOD, POD, CAT) and compatible solutes (proline, glycinebetaine, phenolic contents), which lead to improved gas exchange attributes and water relations and in that way enhance the calcium and potassium contents or decline the sodium and chloride contents in cucumber leaves. Furthermore, triacontanol feeding also shows the answer to yield traits of cucumber. It was concluded from the results that the salinity tolerance efficacy of triacontanol is valid in enhancing the productivity of cucumber plants under salt stress. Triacontanol was more pronounced in green long and marketer green than in summer green and 20252. Hence, the findings of this study pave the way towards the usage of triacontanol @ 0.8 mg L^−1^, and green long and marketer genotypes may be recommended for saline soil.

## Introduction

Almost 7.0% of the world’s total area is affected by salinization^1^, representing more than 900 million hectares of land of the world affected by both saline and sodic conditions^2^, which covers 20% of the cultivated land and half of the irrigated area subjected to high salt levels^3^. Salt affected soils are a significant problem in Pakistan’s agriculture^4,5^. Of that total, approximately 6.3 million hectares or 14% of the irrigated land is now affected by salinity^6^.

Abiotic stresses alter the response of plant growth hormones, resulting in reduced growth and final yield of crop plants^7^. Once plants are subjected to salinity stress, the balance between the production of reactive oxygen species (ROS) and the quenching action of antioxidant enzymes becomes upset, and the resulting plants experience oxidative stress^8^. Plants that have higher concentrations of antioxidant enzymes, either constitutive or induced, have healthy resistance to oxidative damage; for example, superoxide dismutase (SOD), guaiacol peroxidase (POD), catalase (CAT), ascorbate peroxidase (APOX) and glutathione reductase (GR) enhance salt tolerance in plants under saline stress^9^. Additionally, phytohormones have been reported to play a regulatory role in plant growth and development, programmed cell death and survival under changing environmental conditions^10^. Triacontanol is a saturated primary alcohol and component of plant epicuticular waxes with plant growth promoting properties^11,12^ and shows growth stimulating properties at low concentrations^13^, and its foliar application produced significant positive changes in plant photosynthetic pigments, solute accumulation, growth and biomass production under salt stress conditions^14,15^. Foliar feeding of triacontanol induced stress tolerance^16^, by regulating the activities of many antioxidant enzymes^17^, increased the level of osmoprotectants in plant leaf tissues^11,18,19^, and improved the uptake of essential minerals K^+^ and Ca^2+^ instead of Na^+^^14^. In addition, triacontanol produced more reducing sugars, soluble proteins and amino acids under stressed environments^20–22^.

Vegetables are a rich source of phytochemicals and nutrients, which are essential for many metabolic actions in the human body^23^, and vegetable production is endangered by rising soil salinization, predominantly in irrigated croplands, which produce 40.0% of the world’s food requirements^24^. Vegetable crops such as onions, cucumbers, eggplants, peppers, and tomatoes are sensitive to salinity^25^, and cucumber is an important vegetable for human nutrition worldwide^26^. Salt stress had a significant consequence on the growth rate of cucumber; however, salinity levels higher than 25 mM caused a decline in yield up to 13%^27,28^. Deleterious effects of salinity on cucumber lead to decreased plant growth and productivity^5,29,30^. Hence, the current investigation was conducted with the aim of appraising the role of triacontanol in alleviating saline stress in cucumber genotypes and evaluating the ability of triacontanol to stimulate plant growth and productivity. Such information is also key for suggesting a suitable strategy for salt-affected soils. Furthermore, we determined the variations in ionic homeostasis of cucumber genotypes in response to NaCl stress.

## Materials and methods

The current study was conducted under the lath house conditions (30–35 °C and relative humidity 40–50%) of the Institute of Horticultural Sciences, University of Agriculture, Faisalabad, Pakistan (31° 25′ 05.10″ N, 73° 04′ 39.27″ E). It has been confirmed that the experimental samples of plants, including the collection of plant material, complied with relevant institutional, national, and international guidelines and legislation with appropriate permissions from Institute authorities of Institute of Horticultural Sciences, University of Agriculture, Faisalabad Pakistan for collection of plant specimens. In advance of the experiment, the cucumber seed surface was sterilized in a 3% solution of sodium hypochlorite for ten minutes and then properly washed with distilled water and air-dried at 25 °C. Four contrasting cucumber genotypes (Green long, Marketmore, Summer green and 20252)^31^, with varying degrees of salinity tolerance, were used for this experiment. Seeds were sown in plastic 14-L pots in quartz sand with a pH of 6.0–6.5, a field capacity of 7.20% and incipient wilting at a 1.20% volume basis. Four treatments, i.e., *T*0 = control (nonsaline + deionized water spray), *T*1 = (saline + deionized water spray), *T*2 = triacontanol spray (0.80 mg L^−1^) and *T*3 = triacontanol + saline (0.80 mg L^−1^ + NaCl 50 mmol L^−1^) were applied with four replicates. After ten days of emergence, two plants per pot were maintained by thinning out extra plants. Half strength of Hoagland and Arnon (1950)^31^ solution was used as a nutrient source. Salt stress was based on NaCl (MERCK, CAS #-7647-14-5), and NaCl was applied after fifty days of seed emergence in the form of salt solution. Moreover, salt stress was developed at intervals to avoid the osmotic shock of sodium chloride by adjusting 10 mmol L^−1^on Ist day and then gradually increasing after one day intervals until the desired salt level (50 mmol L^−1^) was achieved^31^. The levels of salinity were maintained throughout the experiment by recording the EC and pH of the growing media. Foliar spray of triacontanol was performed using a hand sprayer pump with a full cone nozzle; an already optimized dose of triacontanol (0.8 mg L^−1^) was sprayed after 72 h of stress imposition, while the 2nd spray was applied at the flowering stage and the 3rd spray was applied at the fruit maturing stage. Tween-20 (0.1%) was added to the spray solution as a surfactant to ensure the maximum absorption of the triacontanol solution in the plant tissue. The following data were recorded to evaluate the potential role of triacontanol application.

Stomatal conductance (gs)*,* photosynthetic activity (*Pn*) and transpiration rate (*E*) were measured with a portable apparatus termed IRGA (Analytical Development Company, Hoddesdon, England). All readings of the abovementioned physiological attributes were taken at daytime from 11.00 to 12.00 a.m. by describing a method of^32,33^. SOD activity was examined according to the described procedure of Giannopolitis and Ries^34^. Peroxidase (POD) and catalase (CAT) activities were calculated by the method of Chance and Maehly^35^. For the estimation of chlorophyll contents, a meter model (SPAD-502, Konica Minolta Sensing, Inc; Japan) was used to determine the greenness of cucumber plant leaves. Measurements were taken from fully expanded third to fourth youngest leaves from the apex^36^. Plant cell membrane leakage was calculated using an EC electrical conductivity meter by following the procedure of Lutts^37^. The proline contents were recorded by the described procedure of Bates et al.^38^ and Glycine betaine Grieve and Gratan’s^39^ method. Total phenolic contents were calculated by the described methodology of Julkenen^40^ by using a spectrophotometer (Model: Hitachi-120, Japan).

Leaf water potential (− Ψw) was measured by a pressure chamber (Model 1000, PMS Instrument Co., Albany, NY, USA) to observe that Ψw data were recorded in the early morning before sunrise (6.00 am). For leaf osmotic potential (− Ψs), the same leaf that was used in a pressure chamber for Ψs was placed in a zipper bag and kept at − 80 °C for one week. Then, the leaf material was thawed at room temperature for 30 min, a disposable syringe was used to extract the cell sap, 10 μL of sap was placed on the sensor of the osmometer (Wescor Model-5500) with a disposable syringe, and readings were noted. Leaf turgor potential (Ψp) is measured by the difference between water (Ψw), and osmotic (Ψπ) potential is known as turgor potential (Ψp). Therefore, Ψp was calculated by the following equation: Ψp = Ψw − Ψπ. Leaf relative water contents (LRWC) recorded by the reported method of Wheatherly and Barrs^41^. LRWC (%) = [(FW − DW)/(TW − DW)] × 100. Leaf Na^+^, K^+^ and Ca^+^ were determined by a flame photometer (Jenway PFP-7, UK) by the described procedure of Yoshida et al*.* For chloride (Cl^−^) determination, leaf material was oven-dried at 65 °C and ground to form a powder. This ground dry leaf material (1 g) was heated in a test tube overnight in 20 ml distilled water at 65 °C in an oven. After obtaining the extract, it was filtered with Whatman-40 filter paper and used for the estimation of Cl^−^ ions with a chloride analyzer (Corning-920; Germany). Total soluble solids (TSS) were estimated by using a digital refractometer (PR-32α, ATAGO, CO; LTD. Tokyo, Japan) that gives the °Brix value, and this method was described by^42^.

### Statistical analysis

The experiment was designed with CRD factorial. Analysis of variance (ANOVA) and multiple comparison test (Tukey test) were computed using Statistix 8.1 computer packages. Differences among treatments were considered significant only when a value was lower than *P* ≤ 0.05 after statistical analysis.

## Results

### Stomatal conductance, photosynthesis rate, transpiration rate and water use efficiency

Data for photosynthetic attributes (gs, Pn, E) significantly varied due to NaCl-based salinity and foliar spray of triacontanol (Table 1). A substantial reduction in stomatal conductance was noted in all cucumber genotypes exposed to salt stress. Salt-sensitive genotypes showed a drastic reduction in this gas exchange attribute and resulted in the least decline exhibited by tolerant genotypes; they maintained better conductance of stomata under the saline regime. Foliar application of triacontanol significantly improved stomatal conductance under stressed and nonstressed conditions. Under salinity, the maximum improvement in stomatal conductance was exhibited by Marketmore, followed by Green long in response to foliar-applied triacontanol. On the other hand, transpiration and photosynthetic rate were significantly suppressed in all tested cucumber genotypes due to NaCl stress (Table 1). The summer green genotype showed a better rate of transpiration and photosynthetic rate in the non-saline control but failed to maintain them under salt stress conditions and expressed minimum values for these gas exchange attributes. Foliar application of triacontanol significantly overcomes the lethal effects of salt stress and ameliorates the gas exchange properties of cucumber genotypes. Among the genotypes, Marketmore efficiently responded to exogenously applied triacontanol and resulted in an improved transpiration rate and rate of photosynthesis. Similar to gs, E and Pn, the water use efficiency of cucumber genotypes was also affected by NaCl-based salinity. Summer green and 20252 genotypes showed minimum WUE under stressed conditions, while they showed little improvement with triacontanol, while Marketmore performed well and showed maximum WUE in response to salt stress. Cucumber genotypes subjected to foliar spray of triacontanol significantly improved WUE; however, green long gave higher value for WUE under control and salinity as well and followed by Marketmore.

**Table 1:** Effect of triacontanol on leaf gas attributes of four cucumber genotypes under normal and salt stress conditions.

Treatments	Green long	Marketmore	Summer green	20252	Green long	Marketmore	Summer green	20252
	*Stomatal conductance* (μmol m^−2^ s^−1^)				*Photosynthesis rate* (μmol m^−2^ s^−1^)			
Control	5.25 ± 0.21cd	5.49 ± 0.21bc	5.27 ± 0.19de	5.56 ± 0.29bc	3.57 ± 0.03bc	3.81 ± 0.05bc	3.27 ± 0.23cd	3.72 ± 0.07bc
Salinity (50 mmol L^−1^)	3.87 ± 0.20fg	4.27 ± 0.13gh	2.66 ± 0.10j	2.90 ± 0.34ij	2.79 ± 0.027def	2.82 ± 0.13de	1.90 ± 0.02g	2.09 ± 0.03ef
Triacontanol spray + non-saline	5.90 ± 0.21ab	6.07 ± 0.22a	5.74 ± 0.18bc	6.13 ± 0.27abc	4.12 ± 0.11ab	4.52 ± 0.18a	3.68 ± 0.10bc	4.22 ± 0.23ab
Triacontanol spray + salinity	4.56 ± 0.22de	4.64 ± 0.12ef	3.65 ± 0.38hi	3.74 ± 0.45gh	3.21 ± 0.11cd	3.28 ± 0.23cd	2.36 ± 0.04efg	2.81 ± 0.24de
	*Transpiration rate* (mmol H_2_O m^−2^ s^−1^)				*Water use efficiency* (Pn/E)			
Control	2.64 ± 0.02abc	2.78 ± 0.03abc	2.64 ± 0.05abc	2.86 ± 0.01abc	1.65 ± 0.04ab	1.52 ± 0.04ab	1.31 ± 0.04b	1.37 ± 0.05b
Salinity (50 mmol L^−1^)	2.04 ± 0.12cde	2.04 ± 0.15def	1.59 ± 0.16ef	1.67 ± 0.24f	1.29 ± 0.09b	1.34 ± 0.11ab	0.92 ± 0.12b	0.94 ± 0.20b
Triacontanol spray + non-saline	2.90 ± 0.23abc	3.1 ± 0.29ab	2.86 ± 0.14abc	3.20 ± 0.15a	1.68 ± 0.07a	1.47 ± 0.22ab	1.36 ± 0.05ab	1.22 ± 0.07b
Triacontanol spray + salinity	2.4 ± 0.06bcd	2.49 ± 0.13abcd	2.14 ± 0.12cdef	2.17 ± 0.20cde	1.35 ± 0.02ab	1.29 ± 0.08b	1.09 ± 0.06b	1.20 ± 0.11b

Data represent the means ± SE of four repeats.

Means with different letters are significantly different at *P* ≤ 0.05 according to Tukey’s HSD test (Salt tolerant, Green long and Marketmore) (Salt sensitive Summer green and 20252).

### Antioxidant enzyme activity

**Figure 1:**
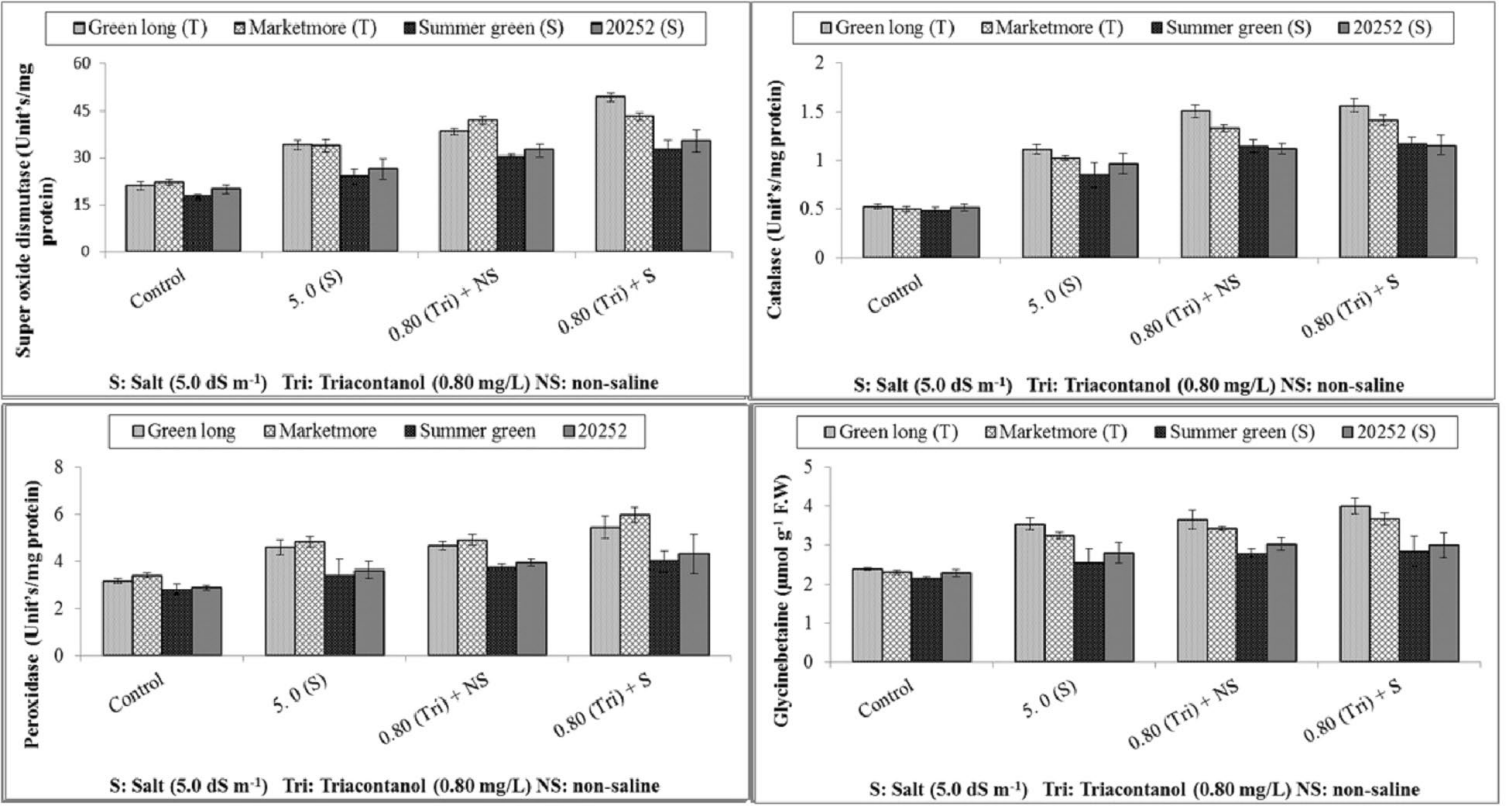
Effect of triacontanol on leaf antioxidants and osmoprotectants of four cucumber genotypes under normal and salt stress conditions.

Figure 1 reveals the effects of foliar spray of triacontanol and salinity treatment on the activities of the studied antioxidants in cucumber genotypes. SOD activity considerably increased with salt stress, especially in green long and marketer, whereas summer green and 20252 showed little increase in SOD activity under stressed and nonstressed environments. Exogenously applied triacontanol caused a further increase in SOD activity in green long followed by marketmore, while summer green showed the minimum value for SOD in response to triacontanol application. Similar to SOD, the POD and CAT activities were also significantly enhanced in all cucumber genotypes exposed to salt stress. However, Green long presented maximum activities of CAT in response to salt stress and foliar applied triacontanol, followed by Marketmore. Likewise, the higher scavenging activity of POD was also noted for Marketmore, while Summer green expressed the lowest values of POD in their leaves under all experimental conditions.

### Estimation of organic solutes

**Figure 2:**
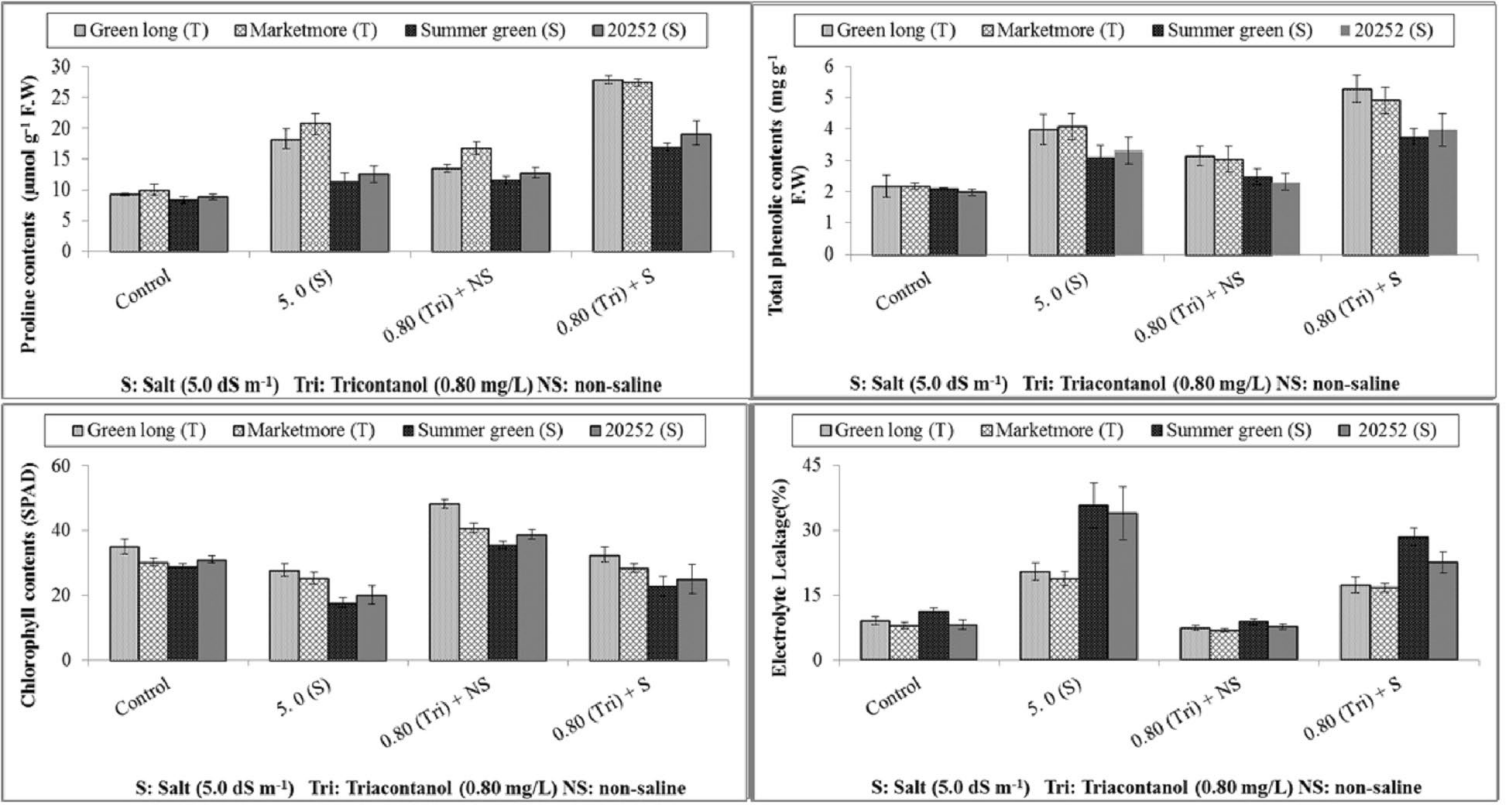
Effect of triacontanol on leaf osmoprotectants and physiological attributes of four cucumber genotypes under normal and salt stress conditions.

The addition of salinity stress increased the accumulation of osmoprotectants (glycine betaine (GB) and proline (Pro) and antioxidant phenols in all cucumber genotypes. Green long revealed high values of GB and Pro contents by significantly enhancing the endogenous levels under saline regime. All the cucumber genotypes countered the foliar spray and improved the contents of GB and pro; however, long-term green treatment with exogenously applied triacontanol significantly increased the GB and Pro contents and showed maximum values for these attributes. On the other hand, the poor response of Summer green and 20252 to foliar spray of triacontanol was observed and resulted in lower values of GB and Pro, respectively, under salt stress (Table 2 & Fig. 2). In the case of total phenols (TP), a similar trend was found, where green long significantly accumulated the highest total phenolics, followed by marketmore supplied with foliar triacontanol under all growing environments. However, minimum accumulation of TP content was noted in summer green under saline stress and triacontanol treatment.

**Table 2:** Effect of triacontanol on leaf water relations of four cucumber genotypes under normal and salt stress conditions.

Treatments	Green long	Marketmore	Summer green	20252	Green long	Marketmore	Summer green	20252
	*Water potential* (Ψw) (− MPa)				*Osmotic potential* (Ψπ) (− MPa)			
Control	0.44 ± 0.04def	0.44 ± 0.03ef	0.54 ± 0.04cde	0.46 ± 0.05def	0.72 ± 0.04efg	0.62 ± 0.04fg	0.84 ± 0.05cde	0.77 ± 0.05efg
Salinity (50 mmol L^−1^)	0.73 ± 0.05bcd	0.64 ± 0.04bcd	1.03 ± 0.10a	0.85 ± 0.10ab	1.08 ± 0.04cde	0.99 ± 0.06cdef	1.67 ± 0.10a	1.50 ± 0.14ab
Triacontanol spray + non-saline	0.38 ± 0.05ef	0.39 ± 0.05ef	0.57 ± 0.03bcd	0.35 ± 0.03f	0.64 ± 0.05fg	0.54 ± 0.03g	0.80 ± 0.05cdef	0.69 ± 0.04efg
Triacontanol spray + salinity	0.63 ± 0.05bcd	0.54 ± 0.04cde	0.84 ± 0.05ab	0.75 ± 0.08abc	0.95 ± 0.10cdef	0.88 ± 0.05cdef	1.24 ± 0.13bc	1.18 ± 0.16bcd
	*Turgor potential* (Ψp) (MPa)				*Leaf relative water contents* (%)			
Control	0.39 ± 0.02bcd	0.35 ± 0.01cde	0.32 ± 0.01def	0.33 ± 0.01def	77.5 ± 3.93ab	81.25 ± 2.21a	73.25 ± 5.11abc	75 ± 3.87abc
Salinity (50 mmol L^−1^)	0.29 ± 0.01fg	0.20 ± 0.02ghi	0.16 ± 0.01i	0.18 ± 0.00hi	59.5 ± 4.11bcd	58 ± 2.48cde	46 ± 5.85e	43.75 ± 5.07e
Triacontanol spray + non-saline	0.51 ± 0.02a	0.45 ± 0.01ab	0.41 ± 0.01bcd	0.43 ± 0.01abc	81 ± 1.47a	83.75 ± 1.89a	75.25 ± 0.85abc	77.25 ± 1.11ab
Triacontanol spray + salinity	0.39 ± 0.019bcd	0.30 ± 0.023ef	0.26 ± 0.019 fgh	0.28 ± 0.012fg	67.25 ± 3.64abc	70.75 ± 2.46abcd	58.5 ± 5.11cde	55.5 ± 7.27de

Data represent the means ± SE of four repeats.

Means with different letters are significantly different at *P* ≤ 0.05 according to Tukey’s HSD test (Salt tolerant, Green long and Marketmore) (Salt sensitive Summer green and 20252).

### Photosynthetic pigments and electrolyte leakage

The results showed that the chlorophyll content of cucumber genotypes was considerably influenced by salt stress. The greenness of cucumber genotypes decreased under salt stress, and a maximum reduction was observed in summer green. In contrast, long green fruits maintained better chlorophyll contents upon exposure to salinity (Fig. 2). All cucumber genotypes effectively enhanced chlorophyll contents upon treatment with foliar-applied triacontanol. Among the hybrids, green long produced maximum chlorophyll contents, while the lowest value was recorded for salt-sensitive summer green, which was revealed by foliar spray of triacontanol in saline environments. Salt stress remarkably affects membrane stability in cucumber genotypes (Fig. 2). Maximum electrolyte leakages were observed in hybrid 20252 followed by Marketmore, whereas Green long explored least electrolyte leakage, which indicates its higher membrane integrity under non-saline controlled conditions. Upon exposure to salinity, Marketmore treated with foliar triacontanol showed maximum membrane stability with low electrolyte leakage compared to other genotypes (Fig. 2).

### Water relations

Exogenously applied triacontanol significantly affected the water relations of the four cucumber genotypes under the NaCl stress regime (Table 2). The maximum reduction in osmotic and water potentials was recorded in the summer green genotype; however, Marketmore secured the best water potential when salt stress was imposed. Likewise, a pattern was observed when these genotypes were supplied with foliar sprays of triacontanol compared to untreated plants. The addition of salt stress significantly reduced the turgor potential in the leaves of all the genotypes. However, foliar feeding of triacontanol improved turgor potential in salt-stressed plants compared to their respective controls. The cucumber genotype Green maintained the highest turgor pressure, followed by Marketmore influence by salt stress and triacontanol. However, summer green results in lower values of turgor potential when subjected to triacontanol under all the experimental conditions. In the case of RWC, higher values were noted for Marketmore and Green long in response to foliar application of triacontanol under stressed and nonstressed environments (Table 2). Under salinity, genotype 20252 leaves showed a maximum reduction in RWC and maintained a minimum RWC when sprayed with triacontanol under controlled and NaCl-stressed conditions.

### Inorganic osmolytes

**Figure 3:**
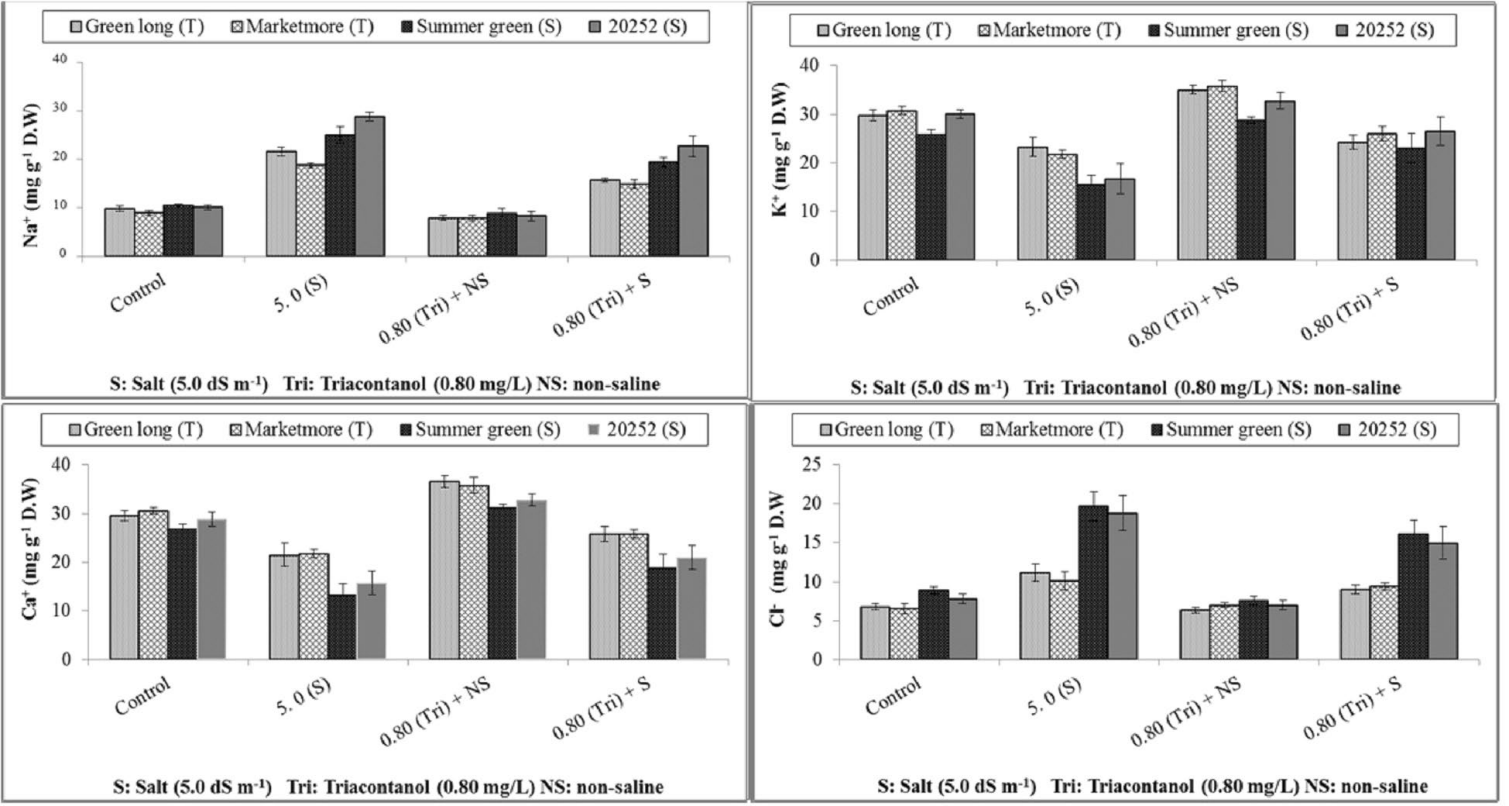
Effect of triacontanol on leaf inorganic osmolytes of four cucumber genotypes under normal and salt stress conditions.

Of many different physiological attributes, leaf ionic contents are a very important feature that reveals the health status of plants and is linked to plant water availability. Salt stress resulted in decreased K^+^ and Ca^+^ contents accompanied by a significant increase in Na^+^ and Cl^−^ contents in the leaves of all cucumber genotypes compared to the non-saline control (Fig. 3). Among the genotypes, lower levels of leaf Na^+^ and Cl^−^ associated with improved K^+^ and Ca^+^ contents were observed in stressed and nonstressed Marketmore and Green plants long sprayed with triacontanol. Summer green accumulated the lowest Ca^+^ and K^+^ contents, while the highest Na^+ content^ was observed in genotype 20252 under saline stress; however, foliar spray of triacontanol improved the ionic contents of these genotypes compared to the nonsprayed control.

### Quality and yield related attributes

**Figure 4:**
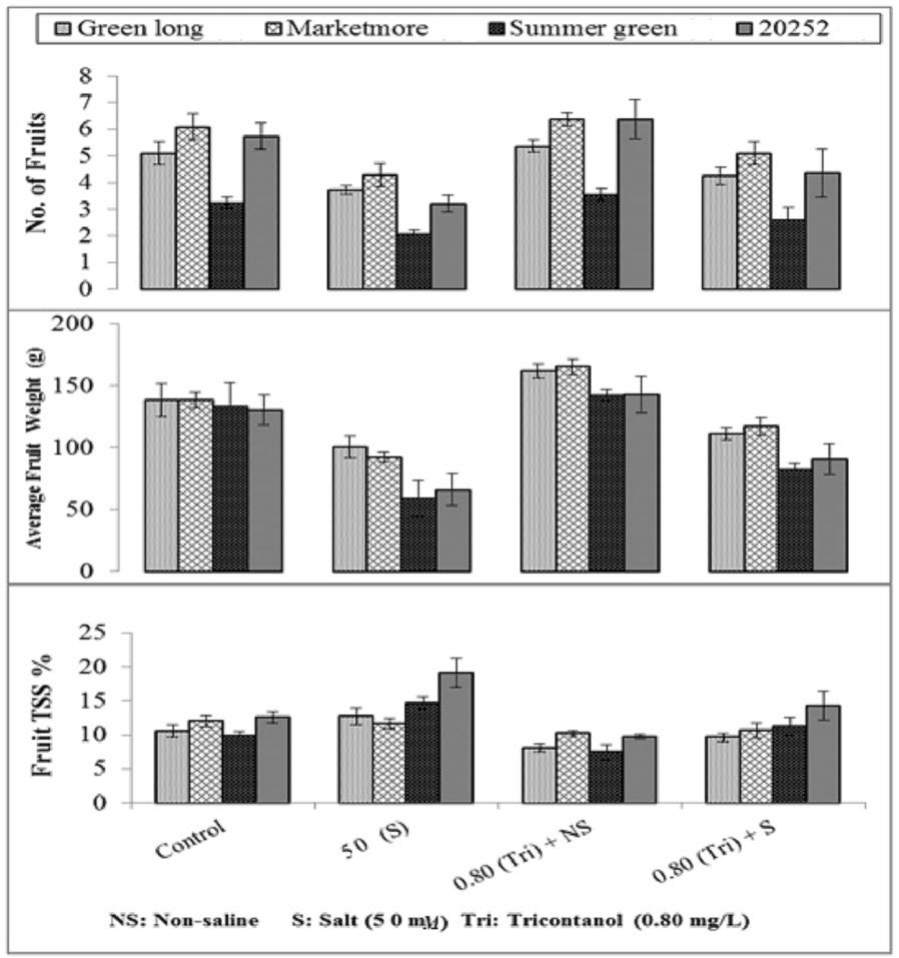
Effect of triacontanol on the yield attributes of four cucumber genotypes under normal and salt stress conditions.

A marked reduction in the total number of fruits and average fruit weight was observed when cucumber genotypes were sown under salt stress (Fig. 4). These reductions were more prominent in the summer green and 20252 genotypes, whereas Marketmore and green long maintained the highest fruit number and weight upon introduction to salinity. Foliar application of triacontanol decreased the adversities of salt stress and improved the no. of fruits and their weight in all evaluated genotypes. However, Marketmore treated with triacontanol showed the maximum number of fruits having high average weight compared to untreated plants under stressed and controlled conditions. Summer green and 20252 showed maximum soluble solids in cucumber fruit and gave poor performance compared to genotypes Marketmore and Green long, which exhibited the lowest concentrations of total soluble solids.

## Discussion

This study was conducted to assess the impacts of an optimized dose of triacontanol (0.80 mg L^−1^) on salt tolerance induction in cucumber genotypes by exploring detailed physiological, biochemical, ionic and yield parameters. Salt stress significantly inhibited the gas exchange mechanisms, while triacontanol (0.80 mg L^−1^) successfully alleviated the salt stress effect. Furthermore, triacontanol improved gas exchange attributes in both salinized and nonsalinized plants of the investigated cucumber genotypes. The ameliorating effect of triacontanol may be attributed to its role in regulating growth hormones (ABA, IAA and cytokinin) because IAA and cytokinin maintain cell division in the apical root meristem, while ABA helps in the biosynthesis of various antistress proteins, strengthens the antioxidant enzyme system and reduces ROS production, maintains membrane integrity and regulates stomatal conductance, which in turn ensures more CO_2_ availability to leaf mesophyll cells, thus improving photosynthetic activity, transpiration rate and water use efficiency. Similar results were reported by many investigators who have explored the effects of triacontanol on several basic metabolic processes, including photosynthesis, nutrient uptake and enzymatic activities^17^. Photosynthetic capacity depends on photosynthetic green pigments such as chlorophyll, and salinity induces a decline in photosynthesis that can be attributed to a reduction in chlorophyll content^43^. Here, it was reported that an accumulation of chlorophyll contents was significantly induced after triacontanol application. These findings are similar to the results of^14^. Triacontanol spraying on leaves significantly enhanced the growth, stomatal conductance, photosynthetic activity, transpiration rate and chlorophyll contents, while under salt stress conditions the membrane permeability reduction has been reported. Triacontanol increases the antenna pigment levels and in PS-II the efficiency of energy has been found to be increased. It has been found that the application of triacontanol showed deep effects on photosynthesis, which leads to increase the plant growth through improvement in overall photosynthesis along with the higher accumulation of organic compounds produced through photosynthesis^44^. It has also been found that the transcription of rbcS gene showed linkage with improved photosynthetesis in plants treated with triacontanol. The triacontanol has been found to be involved in improving the activity of ribulose-1,5-bisphosphate carboxylase oxygenase (RuBisCO) which is an important photosynthetic enzyme due to which the photosystems showed enhanced performace^45^. The application of triacontanol even in very low concentrations enhanced the CO_2_ uptake by plants^46^. Our current study, revelaed that the application of triacontanol was found to be involved in increasing stomata conductance and photosynthesis in cucumber genotypes. The stomata conductance is actually the factor which regualtes photosynthetic rate through regulating the concentration of CO_2_ uptake by leaf mesophyll tissue which is positively and significantly correlated with photosynthetic activity^47^. It has also been reported that triacontanol has positive and significant effects for photosynthetic rate, stomata conductance, and the CO_2_ concentration in leaves of crop plants. Similar, findings were stated by^48,49^. However, salt-tolerant genotypes gave a more efficient response to triacontanol (0.80 mg L^−1^) application than susceptible genotypes under saline (50.0 mm L^−1^) and nonsaline environments. All the water relation attributes (water potential, osmotic potential, turgor potential and leaf relative water contents) were drastically affected by salt stress. In the current experiment, salt stress increased total free proline and glycinebetaine in all genotypes (Green long, Marketmore), and Summer green, 20252) accumulated higher proline. As a positive correlation was found between leaf proline and glycinbetaine contents and leaf water potential (Ψw), which became more negative due to decreased osmotic potential, it could be suggested that these two osmotica play a central role in osmotic adjustment under saline stress^50,51^. Salinity has been known to disturb the water relations of plants as a consequence of decreased leaf osmotic potential in external soil solutions^52^. In our study, leaf Ψw and Ψs decreased under the saline regime. Early responses to salinity included a decrease in water potential and LRWC^53^, and it was observed from the results that plants sprayed with triacontanol @ 0.80 mg L^−1^ showed an improvement in the activities of enzymes such as SOD, CAT and POD under a saline environment (50 mm L^−1^). However, tolerant genotypes exhibited an excellent response in terms of higher antioxidant activities, i.e., SOD, CAT and POD, than sensitive genotypes^54^.

Triacontanol significantly eliminated the drastic effect of salt stress by minimizing ROS with the help and rise of antioxidant enzymes. through the activation and strengthening of the antioxidant system, i.e., SOD and CAT, during osmotic stress because of the accumulation of toxic ions Na^+^ and Cl^−^ and help sustain growth. Thus, the relationship between triacontanol and antioxidants, such as SOD, was reported in the present study^54–56^. Membrane stability under saline stress is an indication of salt stress tolerance. However, excessive accumulation of salt (Na^+^) in the cytosol directly affects membrane stability through enhanced leakage of electrolytes, thereby disturbing cytosolic metabolic activities. It affects physiological and biochemical aspects together with the overproduction of ROS^57^, which leads to premature senescence and a decline in carbon assimilation and, consequently, plant productivity. However, triacontanol foliar spray reduces oxidative stress by enhancing the production of antioxidants, which in turn scavenges ROS and improves carbon assimilation by reducing electrolyte leakage, as observed in the present study, in which triacontanol spray reduced electrolyte leakage. Nevertheless, (Green long and Marketmore) presented better results than (Summer green and 20252) genotypes. Plants may accumulate compatible solutes such as glycinebetaine and proline under salt stress to enhance their salt stress tolerance^58,59^. In this experiment, salinity enhanced glycinebetaine and proline. Phenolics are potential antioxidant compounds that play an important role in scavenging singlet oxygen (O_2_^−^)^60^; under salt stress, the concentrations of phenolic compounds are frequently altered^61^. In this study, saline stress increased the amount of total phenolics in all genotypes. However, foliar-applied triacontanol improved the total phenolic content in tolerant genotypes by presenting a greater degree of improvement. Similar results were reported by^62^.

Foliar application of triacontanol significantly increased the K^+^ and Ca^+^ contents of the leaves. The findings of the present study demonstrated the significant accumulation of mineral contents of K^+^ and Ca^+^ in cucumber. Similar findings were also reported: triacontanol application stimulates K^+^, Ca^+^, and accumulation by eliciting a secondary messenger, L(+) adenosine, and such raised mineral contents may stimulate plant growth. Increased K^+^ content of the leaves may play a role in stomatal function. Triacontanol-induced increased accumulation of nutrients might result in enhanced crop yield^63,64^. However, exogenous application of triacontanol ameliorated the drastic effects of salt stress by reducing Na^+^ contents and increasing K^+^ contents. Moreover, triacontanol proved to be more effective for salt tolerance because it reported a high percentage decrease in leaf Na^+^ contents and a higher percentage increase in K^+^ contents than nontolerant genotypes, which gave a minimum percentage decrease under nonsaline and saline treatments.

Salt tolerance achieved by triacontanol application may be attributed to its ability to maintain enhanced uptake of K^+^ and restricting loading of Na^+^ salts into xylem while maintaining a high K^+^/Na^+^ ratio in plant tissues. This is probably due to the overexpression of SOS1 (Na^+^/H^+^ antiporter), which moves excess Na^+^ out of the cytosol and helps maintain low cytosolic Na^+^ concentrations in almost all tissues, especially in root epidermal cells, particularly root tips in cells bordering vascular tissues. Mainly, SOS1 confers salt stress tolerance by facilitating Na^+^ efflux from the cytosol to the rhizosphere by (1), increasing Na^+^ storage time in vacuoles and reducing Na^+^ buildup in the cytoplasm (2) and controlling long-distance sodium transport through Na^+^ retrieval among roots and shoots. Triacontanol increased yield per plant, enhanced uptake of nutrients, improved photosynthetic rate and nitrogen fixation, and enhanced the translocation of photosynthates and other metabolites^12,65,66^. Salt stress significantly decreased crop quality and yield^67^. Similar findings were reported during our studies; however, triacontanol foliar feeding improved the quality and crop yield^11,64^ because triacontanol application enhances water uptake, cell division, cell elongation and the permeability of plant cell membranes^67^, increased the growth, number of inflorescences, and quality of fruits and increased the yield of the majority of annual vegetables and agronomic crops^20,66,68–71^.

Foliar application of Triacontanol induces the second messenger (TRIM) formation which triggers the infux of ions such as Mg^2+^, K^+^ and Ca^2+^, through opening of their channels in the plasma membrane. Particularly, the level of Ca^2+^ may control for calmodulin and of Mg^2+^ and K^+^ ions which may improve the metabolism of cell by activation of enzymes that may be responsible for plant growth. Modulation of transcription factors such as CAMTA3, GTL and MYB2, that may regulate many anabolic processes which are related to genes. So, better growth of Triacontanol treated plants occurs. Triacontanol reduced the lethal impact of salinity by regulating the stress mitigating genes. Foliar application of Triacontanol over-seed treatment technique could be attributed to its prompt availability to plants. Triacontanol improved the salinity tolerance due to the increased antioxidant enzyme activity, accumulation of osmolytes and maintaining membrane integrity, osmotic adjustment and limiting the lipid peroxidation and ROS production. The lower level of triacontanol mainly showed the useful in improving the salinity tolerance in plants.

## Conclusion

An optimized level of triacontanol@0.8 mg L^−1^ alleviated the lethal effect of salt stress on the studied genotypes under salt-stressed and nonstressed environments. However, the effect of triacontanol was more noticeable on Green long and Marketmore. In view of the salt tolerance potential of these genotypes and the efficacy of triacontanol to mitigate the salt stress impact, cultivation of these genotypes along with foliar application of triacontanol may be endorsed in salty soil.
